# Association of Serum Ascorbic Acid and Zinc Levels with Depressive Symptoms in Young Ecuadorian Adults: A Case–Control Study

**DOI:** 10.3390/medsci14060646

**Published:** 2026-10-08

**Authors:** Shirley Bethzabe Guamán Espinoza, Sara María Cantos-Macías, Luis Fabricio Cordova Reyes, Ginger Jamileth Santana-Moreira, Karime Montes-Escobar, Ana Lúcia S. Rodrigues, Aline Siteneski

**Affiliations:** 1 Facultad de Ciencias de la Salud, Universidad Técnica de Manabí, Portoviejo 130105, Manabí, Ecuador; shirley.guaman@utm.edu.ec (S.B.G.E.); sara.cantos@utm.edu.ec (S.M.C.-M.); ginger.santana@utm.edu.ec (G.J.S.-M.); 2 Departamento de Posgrado, Instituto Universitario Italiano de Rosario, Corrientes 720, PB 1er Piso, Rosario S2000CTT, Argentina; 3 Carrera de Medicina, Campus Portoviejo, Pontificia Universidad Católica del Ecuador Sede Manabí, Portoviejo 130105, Manabí, Ecuador; lfcordova@pucesm.edu.ec; 4 Departamento de Formación y Desarrollo Científico en Ingeniería, Facultad de Ingeniería, Ciencia y Tecnología, Universidad Bernardo O’Higgins, Santiago 8370993, Chile; karime.montes@gmail.com; 5 Departamento de Ciencias Agrícolas, Facultad de Ingeniería Agrícola, Universidad Técnica de Manabí, Portoviejo 130105, Manabí, Ecuador; 6 Statistics Department, University of Salamanca, 37007 Salamanca, Spain; 7 Department of Biochemistry, Center of Biological Sciences, Federal University of Santa Catarina, Florianópolis 88040-900, Santa Catarina, Brazil; alsrodri@gmail.com; 8 Dirección de Investigación, Universidad Técnica de Manabí, Portoviejo 130105, Manabí, Ecuador

**Keywords:** depression, ascorbic acid, zinc, young adults, diet

## Abstract

**Background:** This study assessed the association between serum ascorbic acid and zinc levels and depressive symptoms in young Ecuadorian adults. **Methods:** A case–control study was conducted among individuals aged 18–34 years. A total of 88 young adults were included based on PHQ-9 scores: 44 with moderately severe-to-severe depressive symptoms (PHQ-9 ≥ 15) and 44 with lower depressive symptom severity (PHQ-9 < 15). Serum samples were collected to measure ascorbic acid and zinc concentrations. We also collected sociodemographic data, sleep patterns, and self-reported frequency of consumption of vitamin C- and zinc-rich foods. **Results:** Low serum ascorbic acid (<0.30 mg/dL) was observed in 75% of individuals with PHQ-9 ≥ 15 and 43.2% of those with PHQ-9 < 15 (*p* = 0.002). Multivariable analysis showed that each 0.1 mg/dL decrease in serum ascorbic acid (aOR = 1.78; 95% CI: 1.25–2.66; *p* < 0.001) were associated with higher odds of moderately severe-to-severe depressive symptoms. In crude analyses, sleeping less than 6 h (OR = 3.75; 95% CI: 1.47–10.03; *p* = 0.005) and going to bed after midnight (OR = 3.59; 95% CI: 1.48–9.16; *p* = 0.004) were associated with higher odds of moderately severe-to-severe depressive symptoms. Lower self-reported frequency of consumption of vitamin C-rich foods was also associated with higher depressive symptom severity. Zinc levels showed no significant association. **Conclusions:** Our results show that lower serum ascorbic acid levels are significantly associated with depressive symptoms in young adults. Healthy sleep patterns and more frequent consumption of vitamin C-rich foods were more common in young adults with lower depressive symptom severity.

## 1. Introduction

Depression is one of the leading causes of the global burden of disease among young adults [1,2]. Approximately 3.8% of the global population experiences depression, with a higher prevalence among women [3]. The current DSM-5 diagnostic criteria for major depressive disorder (MDD) encompass nine symptoms, with depressed mood and loss of interest or pleasure (anhedonia) being considered the core symptoms [4,5]. Factors associated with MDD in young adults include unhealthy lifestyle behaviors, such as insufficient sleep, physical inactivity, and an unbalanced diet [6,7]. In severe cases, young adults with MDD may be at increased risk of suicidal behavior [8,9]. The nutritional status of the patient influences the risk of onset, severity, and duration of this disorder [10,11].

Zinc deficiency significantly increases the risk and severity of depressive symptoms [12]. Lower plasma zinc levels have been associated with greater severity of depressive symptoms [3,13]. Among patients receiving selective serotonin reuptake inhibitors (SSRIs), higher zinc intake was associated with lower levels of depressive symptoms compared with lower zinc intake [3,12,14]. Furthermore, patients with treatment-resistant depression showed lower zinc concentrations compared to non-resistant patients [12]. A meta-analysis of 17 studies, including 1643 individuals with depression and 804 controls, found an association between lower blood zinc concentrations (serum/plasma) and depression [15]. These findings suggest that zinc concentrations may be associated with the severity of depressive symptoms and may have potential as a biomarker of depression severity [16].

Ascorbic acid (vitamin C) is a vital antioxidant molecule in the brain [17,18]. Humans cannot produce vitamin C endogenously because they lack L-gulono-γ-lactone oxidase, the enzyme required for the final step of ascorbic acid biosynthesis. Consequently, adequate vitamin C levels must be maintained through dietary intake or supplementation [19]. Previous studies have shown significantly lower serum concentrations of vitamin C in patients with depression compared to healthy controls [20,21]. Furthermore, dietary ascorbic acid supplementation over a period of 6 weeks resulted in a significant reduction in depressive symptoms [21]. Further supporting the potential role of ascorbic acid in depression, evidence suggests that its combination with conventional antidepressants may have a synergistic effect, thereby increasing therapeutic efficacy [19,22]. Adequate vitamin C intake, whether through diet or supplementation, may be relevant to the management of patients with depression [23]. However, epidemiological evidence regarding the association between ascorbic acid levels and depressive symptoms remains limited [17].

Previous studies have reported associations between serum ascorbic acid and zinc levels and the severity of depressive symptoms among adults and older adults [15,16,19,22]. Young adults are particularly vulnerable to mood disorders due to factors such as environmental changes and ongoing hormonal and neuroanatomical development of the brain [24,25]. In contrast, dietary patterns in adulthood tend to be more stable, and the maturation of prefrontal cortex circuitry may contribute to greater resilience to mood disturbances [26,27,28]. In this context, this study aimed to compare the serum levels of ascorbic acid and zinc in young Ecuadorian adults exhibiting moderately severe to severe depressive symptoms (PHQ-9 score ≥ 15) with those with lower depressive symptom severity (PHQ-9 score < 15).

## 2. Materials and Methods

### 2.1. The Study Design

This study was an observational case–control study. The screening occurred between January and March 2025, and biological sample collection took place between April and May 2025 at a designated private laboratory. Participants were undergraduate students aged 18 to 34 years from the Technical University of Manabí, Ecuador. Of the total 825 students who completed the screening questionnaire, 93 were eligible and available. During the second-stage recruitment period, participants were invited and enrolled in the case–control study using a non-probability sampling approach based on participant availability. Five participants with missing PHQ-9 items were excluded, resulting in a final analytical sample of 88 participants (44 with PHQ-9 ≥ 15 and 44 with PHQ-9 < 15). The two groups were not matched for age or sex, and all participants underwent biochemical assessment. Cases included 44 participants with moderately severe-to-severe depressive symptoms (PHQ-9 ≥ 15; observed range 15–26), whereas controls included 44 participants with lower depressive symptom severity (PHQ-9 < 15). All participants in the control group had PHQ-9 scores ranging from 0 to 8; therefore, none had scores in the moderate range, defined as 10–14 points.

To reduce the potential for misclassification bias, the PHQ-9 was administered again immediately before blood collection to confirm participants’ current depressive symptom status. The researchers paid the cost of the laboratory sample. Laboratory personnel were blinded to participants’ depression classification. The final analysis examined associations between depressive symptom severity, sociodemographic characteristics, sleep patterns, and serum levels of ascorbic acid and zinc.

### 2.2. Eligibility Criteria and Outcomes

Inclusion criteria were: (1) being aged 18–34 years; (2) being an undergraduate student at the Technical University of Manabí; and (3) providing written informed consent to participate. Exclusion criteria included: (1) self-reported diagnosis of chronic diseases affecting nutritional status (e.g., chronic kidney disease or diabetes mellitus); (2) current use of vitamin or mineral supplementation; (3) incomplete or missing PHQ-9 data; and (4) refusal to participate in blood collection at the laboratory. Depressive symptom status served as the case definition, whereas serum levels of ascorbic acid and zinc were the primary exposures. The analysis examined the associations between study outcomes and sociodemographic characteristics, sleep patterns, and dietary intake of vitamin C and zinc.

### 2.3. Data Sources/Measurement

The independent variables included sociodemographic factors, lifestyle behaviors, dietary intake, and biochemical markers. The sociodemographic characteristics were as follows: age group (18–20, 21–23, or 24–34 years); sex (female or male); marital status (single, married, or in a consensual union); area of residence (urban or rural); monthly family income (less than one minimum wage, one minimum wage, two minimum wages, or three or more minimum wages); and faculty affiliation (Aquaculture and Marine Sciences; Administrative and Economic Sciences; Education; Health Sciences; Human and Social Sciences; Computer Sciences; Veterinary Sciences; Agronomic Engineering; Engineering and Applied Sciences). Four researcher-designed, non-validated questions were assessed: (Q1) usual sleep at night (6–8 h; less than 6 h; self-reported insomnia); (Q2) usual bedtime (before 10:00 p.m.; between 10:00 p.m. and midnight; after midnight); (Q3) self-reported frequency of consumption of vitamin C-rich foods (never or almost never; once, twice, three times, or more than three times a week); and (Q4) self-reported frequency of consumption of zinc-rich foods (same categories as Q3). These questions only describe the frequency of consumption and do not quantify nutrient intake. Serum ascorbic acid (mg/dL) and zinc (µg/dL) were analyzed as continuous variables and were also dichotomized according to the reference criteria established by the clinical laboratory and specified in the study protocol prior to data analysis. These criteria were as follows: ascorbic acid < 0.30 vs. ≥0.30 mg/dL and zinc < 60 vs. ≥60 µg/dL. The 0.30 mg/dL cut-off does not correspond to a universally accepted definition of vitamin C deficiency; therefore, alternative thresholds were examined in sensitivity analyses.

### 2.4. Depressive Symptoms

Depression was considered the dependent variable and was assessed using the Patient Health Questionnaire-9 (PHQ-9) [29]. The PHQ-9 contains nine items rated on a 4-point scale based on the frequency of symptoms experienced over the past 2 weeks [30]. It has demonstrated adequate test–retest reliability (0.84) and internal consistency (α = 0.89) [31]. This study used the Spanish version, which has comparable psychometric properties to the original [29,31]. Each PHQ-9 item was scored on a four-point Likert-type scale ranging from 0 (not at all) to 3 (nearly every day). We selected a PHQ-9 threshold of ≥15 to distinguish participants with moderately severe-to-severe depressive symptoms as opposed to those with mild symptoms. This threshold was chosen to improve the distinction between cases and controls. The clinical diagnosis of depression requires a comprehensive clinical assessment and is not based solely on the PHQ-9. In this study, participants with PHQ-9 scores ≥ 15 were classified as having higher depressive symptom severity, while those with scores < 15 were classified as having lower symptom severity.

### 2.5. Biochemical Markers

All biochemical analyses of the study were performed at Interlab, a private clinical laboratory in Ecuador, with certified quality and technical competence. The laboratory was not involved in the study design, data collection, or interpretation. Each sample was processed upon payment, using internationally standardized protocols under certified quality assurance procedures to ensure analytical accuracy.

Serum ascorbic acid concentrations were measured by chromatography coupled with mass spectrometry and validated in accordance with CLIA regulations by Quest Diagnostics (Chantilly, VA, USA). Blood samples were obtained from fasting participants, who were instructed to avoid fruits and vitamin C supplements for at least 24 h prior to collection. Serum was protected from light, separated within one hour, and immediately frozen at −70 °C. Only samples meeting strict pre-analytical criteria (no hemolysis, no lipemia, no exposure to room temperature) were included in the analysis. The assay showed stability for up to 35 days at −70 °C. The reference ranges were 0.3–2.7 mg/dL for females and 0.2–2.1 mg/dL for males.

Serum zinc was quantified using graphite furnace atomic absorption spectrometry (GFAAS), following standardized procedures in the laboratory. Samples were collected via venipuncture using separator gel tubes and stored at 2–8 °C until analysis. MGA-1000 atomic absorption spectrometer (Lumex Instruments, Mission, BC, Canada) and ultrapure water from Labostar PRO (Evoqua, Germany) were used. Calibration was performed using three certified standards (three certified standards (AccuStandard, Inc., New Haven, CT, USA) at 500, 1000, and 3000 μg/L, with an accepted linearity of R^2^ ≥ 0.995. Each serum sample (10 μL) was combined with 6 μL of 2% phosphate matrix modifier. The reference range for adults (≥18 years) is 60–130 μg/dL.

### 2.6. Ethical Considerations

The Human Research Ethics Committee of the Technical University of Manabí, Ecuador (CEISH-UTM-INT_23-12-14_SBGE) approved the study. Written informed consent was obtained from all study participants.

### 2.7. Statistical Analysis

Participants with moderately severe-to-severe depressive symptoms (PHQ-9 ≥ 15) were compared with participants with lower depressive symptom severity (PHQ-9 < 15). Categorical variables are presented as absolute frequencies and percentages and were compared using Pearson’s chi-square test or, when more than 20% of expected cell counts were below five, Fisher’s exact test (2 × 2 tables) or the Fisher–Freeman–Halton exact test (2 × k tables). We assessed the distribution of continuous variables with the Shapiro–Wilk test; because none were normally distributed, we present continuous variables as median (interquartile range, IQR) and compare them using the Mann–Whitney U test. Serum ascorbic acid values below the laboratory limit of quantification (<0.1 mg/dL) were assigned a value of 0.01 mg/dL, which does not affect rank-based comparisons.

Due to the small sample size and the presence of zero or sparse cells, which resulted in complete or nearly complete separation in conventional maximum-likelihood logistic regression, we used Firth’s penalized-likelihood logistic regression to estimate associations with PHQ-9 ≥ 15. Odds ratios (ORs) are reported with 95% confidence intervals (CIs) based on the profile-penalized likelihood, as well as *p*-values from penalized likelihood-ratio tests. Before modeling, sparse categories were collapsed: marital status (single vs. married/common-law union), monthly family income (less than one, one, or two or more minimum wages), faculty (Health Sciences vs. other faculties), bedtime (before vs. after midnight), frequency of vitamin C-rich food consumption (never or rarely; once or twice a week; three or more times a week) and frequency of zinc-rich food consumption (twice a week or less vs. three or more times a week). Serum ascorbic acid, the primary exposure, was analyzed as a continuous variable (or per 0.1 mg/dL decrease) and, additionally, as a dichotomous variable using the laboratory cut-off (<0.30 vs. ≥0.30 mg/dL). Serum zinc concentrations were analyzed as a continuous variable and additionally dichotomized using a cut-off of 60 µg/dL.

To avoid overfitting, the multivariable models were restricted to four parameters corresponding to approximately 11 events per parameter. These parameters were specified a priori and included serum ascorbic acid, monthly family income (less than one minimum wage vs. one or more minimum wages), faculty (other faculties vs. Health Sciences), and sex. Faculty was included due to the significant imbalance between groups. Model 1 included ascorbic acid as a continuous variable, while Model 2 included it as a dichotomous variable. Sleep variables were not included in the adjusted models because sleep disturbance is one of the nine PHQ-9 items and therefore partly overlaps with the outcome definition. Sensitivity analyses were performed using (i) alternative ascorbic acid cut-offs (<0.20 mg/dL and <0.40 mg/dL for deficiency and hypovitaminosis, respectively); (ii) sex-specific lower reference limits (females < 0.30 mg/dL; males < 0.20 mg/dL); and (iii) additional adjustment for bedtime or serum zinc. A two-sided *p*-value < 0.05 was considered statistically significant. Analyses were performed in Python 3 (NumPy, SciPy, pandas) using an implementation of Firth’s method equivalent to the default settings of the R package logistf (profile-penalized likelihood confidence intervals and penalized likelihood-ratio tests). The results of the sensitivity analyses are provided in Supplementary Table S1.

## 3. Results

Table 1 shows the sociodemographic characteristics of the students. PHQ-9 scores ranged from 15 to 26 (median 20) in the higher-severity group and from 0 to 8 (median 3) in the lower-severity group. Age and sex distributions were similar between groups (*p* = 0.846 and *p* = 1.000, respectively). Monthly family income differed between groups (*p* = 0.002): 63.6% of participants with PHQ-9 ≥ 15 reported an income below one minimum wage, compared with 25.0% of those with PHQ-9 < 15. Faculty distribution also differed significantly (*p* < 0.001), with Health Sciences students accounting for 77.3% of participants with PHQ-9 < 15 and 29.5% of those with PHQ-9 ≥ 15. Sleep patterns differed between groups (*p* < 0.001): self-reported insomnia was only reported by participants with PHQ-9 ≥ 15 (29.5% vs. 0%). Regarding sleep habits, 45.5% and 31.8% reported sleeping less than 6 h, respectively. Going to bed after midnight was more frequent in the higher-severity group (52.3% vs. 22.7%; *p* = 0.007). Never or rarely consuming vitamin C-rich foods was reported by 45.5% and 6.8% of participants, respectively (*p* < 0.001), whereas zinc-rich food consumption did not differ significantly (*p* = 0.051). Median serum ascorbic acid was lower in participants with PHQ-9 ≥ 15 than in those with PHQ-9 < 15 [0.10 (IQR 0.10–0.28) vs. 0.30 (IQR 0.12–0.40) mg/dL; *p* < 0.001], and 75.0% vs. 43.2% had concentrations <0.30 mg/dL (*p* = 0.002). Serum zinc did not differ between groups, either as a continuous variable [90.45 (IQR 78.26–100.40) vs. 85.44 (IQR 72.51–99.17) µg/dL; *p* = 0.341] or as a categorical variable (*p* = 0.676).

In the crude Firth analyses (Table 2), higher odds of PHQ-9 ≥ 15 were observed for family income below one minimum wage (OR = 5.61; 95% CI: 2.07–16.31; *p* < 0.001), those studying in a faculty other than Health Sciences (OR = 7.67; 95% CI: 3.09–20.44; *p* < 0.001), those sleeping less than 6 h (OR = 3.75; 95% CI: 1.47–10.03; *p* = 0.005) compared with 6–8 h, and those going to bed after midnight (OR = 3.59; 95% CI: 1.48–9.16; *p* = 0.004). Higher odds were also observed among participants who never or rarely consumed vitamin C-rich foods compared with those who consumed them three or more times per week (OR = 14.87; 95% CI: 3.83–73.33; *p* < 0.001), those who consumed zinc-rich foods twice a week or less (OR = 3.00; 95% CI: 1.18–8.10; *p* = 0.020), and those with serum ascorbic acid < 0.30 mg/dL (OR = 3.81; 95% CI: 1.59–9.57; *p* = 0.002). Each 0.1 mg/dL decrease in serum ascorbic acid was associated with higher odds of PHQ-9 ≥ 15 (OR = 1.90; 95% CI: 1.38–2.74; *p* < 0.001). In crude analyses, self-reported insomnia was strongly associated with PHQ-9 ≥15. However, the estimate was very imprecise (OR = 71.6; 95% CI: 8.3–9441) because no participant in the lower-severity group reported insomnia. Age, sex, marital status, area of residence, and serum zinc, analyzed as categorical or continuous variables, were not associated with depressive symptom severity.

In the multivariable Firth models (Table 3), lower serum ascorbic acid remained associated with PHQ-9 ≥ 15 after adjustment for family income, faculty, and sex (Model 1: aOR per 0.1 mg/dL decrease = 1.78; 95% CI: 1.25–2.66; *p* < 0.001). Family income below one minimum wage (aOR = 3.09; 95% CI: 1.08–9.14; *p* = 0.035) and studying in a faculty other than Health Sciences (aOR = 4.55; 95% CI: 1.63–13.36; *p* = 0.004) were also associated with higher odds of PHQ-9 ≥ 15, whereas sex was not. When ascorbic acid was dichotomized at the laboratory cut-off (Model 2), concentrations < 0.30 mg/dL were associated with higher odds of PHQ-9 ≥ 15 (aOR = 3.42; 95% CI: 1.26–9.93; *p* = 0.016). Penalization yielded finite estimates for all parameters; the confidence intervals reflect the limited sample size.

In the sensitivity analyses (Table S1), the association between lower serum ascorbic acid levels and PHQ-9 ≥ 15 remained consistent when using a cut-off < 0.20 mg/dL (aOR = 2.82; 95% CI: 1.05–7.94; *p* = 0.040) or sex-specific lower reference limits (aOR = 4.31; 95% CI: 1.58–12.69; *p* = 0.004) were applied, and after additional adjustment for bedtime (aOR = 1.91; 95% CI: 1.29–3.07) or serum zinc (aOR = 1.77; 95% CI: 1.25–2.64). At the <0.40 mg/dL threshold, all participants with PHQ-9 ≥ 15 had concentrations below the cut-off, resulting in a highly imprecise estimate (aOR = 62.9; 95% CI: 6.8–8493). This estimate is reported for completeness only.

## 4. Discussion

Our results show that low serum levels of ascorbic acid were significantly associated with higher levels of depressive symptoms in a population of young undergraduate students. Specifically, in this case–control analysis, individuals with serum ascorbic acid concentrations < 0.30 mg/dL had 3.42 times the adjusted odds of having moderately severe-to-severe depressive symptoms compared to those with serum ascorbic acid levels ≥ 0.30 mg/dL. Socioeconomic status was also significantly associated with depressive symptoms, with participants earning below the minimum wage presenting higher odds of having moderately severe-to-severe depressive symptoms. In contrast, sleeping less than 6 h and going to bed after midnight were associated with higher odds of moderately severe-to-severe depressive symptoms compared with sleeping 6–8 h and going to bed before midnight, respectively. Serum zinc levels, however, were not significantly associated with depressive symptoms. Previous studies have associated zinc with a lower risk of depression, and vitamin C with depressive-like behaviors through monoaminergic mechanisms [32,33].

Lower self-reported frequency of consumption of vitamin C-rich foods was more prevalent among young adults with higher depressive symptom severity. Despite Ecuador’s wide variety of fruits rich in vitamin C and their easy accessibility, frequent consumption (more than three times per week) was limited in young adults [34]. Higher dietary vitamin C intake, particularly from vegetables, is associated with a lower risk of depressive symptoms in the general population, highlighting the potential protective role of vegetable consumption [35]. However, higher vitamin C intake does not seem to significantly contribute to symptom improvement in patients undergoing treatment for depression [36]. A meta-analysis of observational studies revealed an inverse association between dietary vitamin C intake and depression, showing a negative correlation between dietary vitamin C intake and depressive symptoms [17].

In our study, lower self-reported frequency of consumption of vitamin C-rich foods and lower serum ascorbic acid levels were significantly associated with greater depressive symptom severity in young adults. Consistent with previous studies, lower vitamin C intake among younger individuals has been associated with higher depression scores [17,34,35], though a systematic review of clinical trials found no significant improvement in mood status following vitamin C supplementation [36]. Age may significantly influence the role of vitamin C intake on depression severity [37]. For instance, a case–control study conducted in the United States among individuals aged 20–80 years found a negative correlation between serum ascorbic acid concentrations and depression, suggesting that higher ascorbic acid concentrations are associated with a lower prevalence of depressive symptoms [38]. Overall, these findings indicate that age may affect the relationship between vitamin C status and depressive symptoms across the lifespan. Another consideration is the possibility of reverse causality, whereby participants with more severe depressive symptoms may have poorer dietary habits and lower consumption of micronutrient-rich foods, potentially resulting in lower serum vitamin C concentrations. Preclinical studies suggest that ascorbic acid may influence neuroplasticity through glutamatergic neurotransmission and signaling pathways involving NMDA receptors, ERK1/2, PI3K/Akt, mTOR, and BDNF, as well as through mechanisms related to oxidative and inflammatory processes [39,40]. However, these mechanisms were not evaluated in the present study and should therefore be considered potential explanations for the observed association between serum ascorbic acid concentrations and depressive symptom severity.

Previous studies have reported that zinc deficiency correlates with the severity of depressive symptoms in adults with a mean age of 37.7 or 49.4 years [13,41]. However, clinicians generally approach zinc supplementation with caution, as excessive zinc intake may have adverse neurological effects [42]. Greater depressive symptom severity was associated with lower serum zinc concentrations compared with controls [13]. Nevertheless, our results show that neither dietary zinc intake nor serum zinc concentrations were significantly associated with depressive symptoms. The typical diet of young Ecuadorians often includes animal-based foods such as red meat, poultry, and seafood [43]. This discrepancy may reflect differences in study populations, including age, diet, or underlying genetic and ethnic variability in serum zinc levels association with depressive symptoms [44,45,46].

In our study, short sleep duration (<6 h/night) and late bedtimes were significantly associated with higher odds of depressive symptoms, whereas adequate sleep duration (6–8 h/night) and earlier bedtimes were associated with lower odds. The National Sleep Foundation recommends that young adults sleep 7–9 h per night to support optimal mental health. Our results align with previous studies showing that poor sleep quality is associated with higher depression scores in young adults [47]. Sleep disturbances, including insomnia and hypersomnia, have also been associated with cognitive impairment and emotional dysregulation [48,49,50]. Moreover, poor sleep quality has been linked to both the onset and persistence of depressive symptoms [51,52] as well as to greater symptom severity in young adults [53]. Finally, lower family income was more frequent among young adults with moderately severe-to-severe depressive symptoms. This finding is consistent with previous research linking economic stressors to an increased risk of depression, potentially due to reduced access to resources [54,55].

This study has several limitations. First, the study design precludes causal inferences regarding the association between depressive symptoms and serum micronutrient levels. Although we assessed the frequency of consumption of vitamin C- and zinc-rich foods using self-reported questions, the lack of quantitative intake data prevented estimation of nutrient intake. This study used researcher-designed, non-validated dietary questions; therefore, these findings should be interpreted with caution. To assess the frequency of consumption of foods containing vitamin C and zinc, we used a predefined list of commonly consumed foods. Nevertheless, using the PHQ-9, a widely validated and reliable instrument for assessing depressive symptoms, strengthens the consistency and comparability of our findings. Additionally, integrating self-reported data with objective, a private clinical laboratory in Ecuador assessments of serum vitamin C and zinc enhances the study’s internal validity. Important potential confounders, including BMI, smoking, alcohol consumption, physical activity, acute infection/inflammation, oral contraceptive use, and season of sampling, were not systematically collected and could not be included in the adjusted analyses, resulting in potential residual confounding. Even though the relatively small and non-probabilistic sample may limit generalizability, our findings provide important preliminary insight into the relationship between depressive symptoms and serum vitamin C and zinc levels among university students in a Latin American context. This is relevant considering the lack of studies addressing mental health and nutrition in young adults in this region. Future longitudinal and interventional studies are warranted to further investigate the potential relationship between vitamin C status and subsequent mental health outcomes in young adults and to determine whether zinc status may also be relevant to this association.

## 5. Conclusions

This case–control study showed that low serum ascorbic acid levels were more prevalent among young adults with moderately severe-to-severe depressive symptoms. Health-related behaviors, such as sleeping between 6 and 8 h and having earlier bedtimes, as well as more frequent consumption of vitamin C-rich foods, were more common in individuals with lower depressive symptom severity. No significant association was observed between serum zinc levels and depressive symptoms. Additionally, lower income was more frequently reported among participants with moderately severe-to-severe depressive symptoms. Given the limited research on young adults, these findings underscore the significant link between serum ascorbic acid levels and depressive symptom severity, while highlighting the importance of lifestyle and socioeconomic factors in this context.

## Figures and Tables

**Table 1:** Characteristics of participants with moderately severe-to-severe (PHQ-9 ≥ 15) and lower depressive symptom severity (PHQ-9 < 15).

Variable	Moderately Severe-to-Severe (*n* = 44) *n*/Median	%	Lower Depressive Symptom Severity < 15 (*n* = 44) *n*/Median	%	*p* Value
Age (years), median (IQR)	21.00 (19.00–22.00)		20.50 (19.25–22.00)		0.846
**PHQ-9 score, median (IQR)**	**20.00 (15.00–22.75)**		**3.00 (1.00–3.00)**		**<0.001**
**Serum ascorbic acid (mg/dL), median (IQR)**	**0.10 (0.10–0.28)**		**0.30 (0.12–0.40)**		**<0.001**
Serum zinc (µg/dL), median (IQR)	90.45 (78.26–100.40)		85.44 (72.51–99.17)		0.341
**Age group**					0.706
18–20	21	47.7	22	50.0	
21–23	13	29.5	15	34.1	
24–34	10	22.7	7	15.9	
**Sex**					1.000
Female	29	65.9	29	65.9	
Male	15	34.1	15	34.1	
**Marital status**					1.000
Single	42	95.5	41	93.2	
Married	0	0.0	1	2.3	
Common-law union	2	4.5	2	4.5	
**Area of residence**					0.106
Urban	27	61.4	34	77.3	
Rural	17	38.6	10	22.7	
**Monthly family income**					**0.002**
Less than one minimum wage	28	63.6	11	25.0	
One minimum wage	9	20.5	21	47.7	
Two minimum wage	4	9.1	5	11.4	
Three or more minimum wage	3	6.8	7	15.9	
**Faculty**					**<0.001**
Health Sciences	13	29.5	34	77.3	
Educational Sciences	7	15.9	5	11.4	
Humanities and Social Sciences	6	13.6	2	4.5	
Engineering and Applied Sciences	6	13.6	2	4.5	
Administrative and Economic Sciences	4	9.1	0	0.0	
Veterinary Sciences	3	6.8	0	0.0	
Computer Sciences	2	4.5	1	2.3	
Agronomic Engineering	2	4.5	0	0.0	
Aquaculture and Marine Sciences	1	2.3	0	0.0	
**Q1. Sleep duration**					**<0.001**
6–8 h	11	25.0	30	68.2	
Less than 6 h	20	45.5	14	31.8	
Self-reported insomnia	13	29.5	0	0.0	
**Q2. Usual bedtime**					**0.007**
Before 10:00 p.m.	1	2.3	5	11.4	
Between 10:00 p.m. and midnight	20	45.5	29	65.9	
After midnight	23	52.3	10	22.7	
**Q3. Frequency of vitamin C-rich food consumption**					**<0.001**
Never or almost never	20	45.5	3	6.8	
Once a week	13	29.5	13	29.5	
Twice a week	5	11.4	12	27.3	
Three times a week	1	2.3	11	25.0	
More than three times a week	5	11.4	5	11.4	
**Q4. Frequency of zinc-rich food consumption**					**0.051**
Once a week	9	20.5	3	6.8	
Twice a week	9	20.5	5	11.4	
Three times a week	5	11.4	13	29.5	
More than three times a week	21	47.7	23	52.3	
**Serum ascorbic acid**					**0.002**
<0.30 mg/dL	33	75.0	19	43.2	
≥0.30 mg/dL	11	25.0	25	56.8	
**Serum zinc**					0.676
<60 µg/dL	2	4.5	4	9.1	
≥60 µg/dL	42	95.5	40	90.9	

Note: Values are *n* (%) unless otherwise indicated. IQR, interquartile range. Q1, sleep duration; Q2, usual bedtime; Q3, self-reported frequency of vitamin C-rich food consumption; Q4, self-reported frequency of zinc-rich food consumption. Q1–Q4 were researcher-designed, non-validated questions. *p* values were obtained using Pearson’s chi-square test, Fisher’s exact test (2 × 2), or the Fisher–Freeman–Halton exact test (2 × k) when more than 20% of expected counts were <5, and the Mann–Whitney U test for continuous variables. Serum ascorbic acid values below the limit of quantification (<0.1 mg/dL) were set at 0.01 mg/dL. Bold indicates *p* < 0.05.

**Table 2:** Crude associations of sociodemographic characteristics, sleep, frequency of food consumption and serum ascorbic acid and zinc with moderately severe-to-severe depressive symptoms (PHQ-9 ≥ 15): Firth penalized logistic regression.

Variable	OR	95% CI	*p* Value
**Age group**			
21–23	0.91	0.35–2.34	0.847
24–34	1.47	0.49–4.57	0.497
18–20	1 (reference)		
**Sex**			
Female	1.00	0.42–2.40	1.000
Male	1 (reference)		
**Marital status**			
Married/common-law union	0.70	0.11–3.77	0.672
Single	1 (reference)		
**Area of residence**			
Rural	2.09	0.85–5.35	0.110
Urban	1 (reference)		
**Monthly family income**			
Less than one minimum wage	5.61	2.07–16.31	**<0.001**
Two or more minimum wage	1.36	0.41–4.47	0.613
One minimum wage	1 (reference)		
**Faculty**			
Other faculties	7.67	3.09–20.44	**<0.001**
Health Sciences	1 (reference)		
**Q1. Sleep duration**			
Less than 6 h	3.75	1.47–10.03	**0.005**
Self-reported insomnia †	71.61	8.30–9441	**<0.001**
6–8 h	1 (reference)		
**Q2. Usual bedtime**			
After midnight	3.59	1.48–9.16	**0.004**
Before midnight	1 (reference)		
**Q3. Vitamin C-rich foods**			
Never or almost never	14.87	3.83–73.33	**<0.001**
Once or twice a week	1.84	0.64–5.76	0.263
Three or more times a week	1 (reference)		
**Q4. Zinc-rich foods**			
Twice a week or less	3.00	1.18–8.10	**0.020**
Three or more times a week	1 (reference)		
**Serum ascorbic acid (categorical)**			
<0.30 mg/dL	3.81	1.59–9.57	**0.002**
≥0.30 mg/dL	1 (reference)		
Serum ascorbic acid, per 0.1 mg/dL decrease	1.90	1.38–2.74	**<0.001**
**Serum zinc (categorical)**			
<60 µg/dL	0.53	0.09–2.53	0.428
≥60 µg/dL	1 (reference)		
Serum zinc, per 10 µg/dL increase	1.08	0.93–1.28	0.316

Note: OR, odds ratio; CI, confidence interval (profile penalized likelihood). ORs were estimated using Firth penalized logistic regression; *p* values were obtained from penalized likelihood-ratio tests. Categories with zero or sparse cells were collapsed. Q1, “How many hours do you sleep at night?”; Q2, “What time do you usually go to bed?”; Q3, “How often do you eat foods that contain vitamin C (guava, papaya, kiwi, lemon, orange, strawberry, etc.)?”; Q4, “How often do you eat foods that contain zinc (beef, chicken, lamb, guinea pig)?”. † The estimate is imprecise because no participant with PHQ-9 < 15 reported insomnia. Bold indicates *p* < 0.05.

**Table 3:** Multivariable Firth penalized logistic regression models of factors associated with moderately severe-to-severe depressive symptoms (PHQ-9 ≥ 15) in Ecuadorian young adults.

Variable	aOR	95% CI	*p* Value
**Model 1** **—Serum ascorbic acid as a continuous variable**			
Serum ascorbic acid, per 0.1 mg/dL decrease	1.78	1.25–2.66	**<0.001**
Family income < 1 minimum wage (ref. ≥ 1 minimum wage)	3.09	1.08–9.14	**0.035**
Other faculties (ref. Health Sciences)	4.55	1.63–13.36	**0.004**
Female (ref. male)	1.17	0.39–3.50	0.777
**Model 2—Serum ascorbic acid dichotomized at 0.30 mg/dL**			
Serum ascorbic acid < 0.30 mg/dL (ref. ≥ 0.30 mg/dL)	3.42	1.26–9.93	**0.016**
Family income < 1 minimum wage (ref. ≥ 1 minimum wage)	3.16	1.15–8.95	**0.025**
Other faculties (ref. Health Sciences)	5.23	1.96–14.79	**<0.001**
Female (ref. male)	1.14	0.39–3.38	0.812

Note: aOR, adjusted odds ratio; CI, profile penalized likelihood confidence interval; *p* values from penalized likelihood-ratio tests. Outcome reference category: PHQ-9 < 15. Each model includes all variables shown (four parameters; 44 events). Bold, *p* < 0.05.

## Data Availability

The data supporting the findings of this study are available from the corresponding author upon reasonable request.

## Supplementary Materials

The following supporting information can be downloaded at https://www.mdpi.com/article/10.3390/medsci14060646/s1. Table S1: Sensitivity analyses for the association between serum ascorbic acid and moderately severe-to-severe depressive symptoms (PHQ-9 ≥ 15).
