# Supplementary material for: Association of Serum Ascorbic Acid and Zinc Levels with Depressive Symptoms in Young Ecuadorian Adults: A Case–Control Study
Source: Med Sci (Basel). 2026 Oct 8;14(6):646. doi: 10.3390/medsci14060646 (PMC13706711; doi:10.3390/medsci14060646)
Supplement: Supplementary file 1 [file medsci-14-00646-s001.zip › medsci-4593971-supplementary.pdf]

medical sciences

MDPI

Article

Association of Serum Ascorbic Acid and Zinc Levels with Depressive Symptoms in Young Ecuadorian Adults: A Case–Control Study

Table S1. Sensitivity analyses for the association between serum ascorbic acid and moderately severe-to-severe depressive symptoms (PHQ-9 ≥15). All models adjusted for family income, faculty and sex (Firth penalized logistic regression).

Analysis	Below cut-off PHQ-9 ≥15 vs <15	aOR	95% CI	p value
Main analysis (Model 1): per 0.1 mg/dL decrease	—	1.78	1.25–2.66	**< 0.001**
Laboratory cut-off (Model 2): < 0.30 mg/dL	33/44 vs 19/44	3.42	1.26–9.93	**0.016**
Ascorbic acid < 0.20 mg/dL (deficiency)	24/44 vs 11/44	2.82	1.05–7.94	**0.040**
Ascorbic acid < 0.40 mg/dL (hypovitaminosis)‡	44/44 vs 26/44	62.93	6.85–8493	**< 0.001**
Below sex-specific lower limit (female < 0.30; male < 0.20 mg/dL)	29/44 vs 13/44	4.31	1.58–12.69	**0.004**
Per 0.1 mg/dL decrease, additionally adjusted for bedtime after midnight	—	1.91	1.29–3.07	**< 0.001**
Per 0.1 mg/dL decrease, additionally adjusted for serum zinc	—	1.77	1.24–2.64	**< 0.001**

Note. aOR, adjusted odds ratio; CI, profile penalized likelihood confidence interval. ‡All participants with PHQ-9 ≥15 had concentrations < 0.40 mg/dL; the estimate is obtained only because of penalization and is highly imprecise. Bold, $p < 0.05$.
